# Supplementary material for: Clinical trial links oncolytic immunoactivation to survival in glioblastoma
Source: Nature. 2023 Oct 18;623(7985):157–66. doi: 10.1038/s41586-023-06623-2 (PMC10620094; doi:10.1038/s41586-023-06623-2)
Supplement: Supplementary file 1 — Supplementary Methods, Supplementary Notes and Supplementary Fig. 1 (the uncropped gels). [file 41586_2023_6623_MOESM1_ESM.pdf]

nature portfolio

https://doi.org/10.1038/s41586-023-06623-2

Supplementary information

Clinical trial links oncolytic immunoactivation to survival in glioblastoma

In the format provided by the authors and unedited

Supplementary Information (SI): Methods
Clinical trial links oncolytic immunoactivation to survival in glioblastoma

Authors: Alexander L. Ling[1]†, Isaac H. Solomon[2]†, Ana Montalvo Landivar[1]†, Hiroshi Nakashima[1]†, Jared K. Woods[2], Andres Santos[1,2], Nafisa Masud[1], Geoffrey Fell[3], Xiaokui Mo[4,5], Ayse S. Yilmaz[4,5], James Grant[1], Abigail Zhang[1], Joshua D. Bernstock[1], Erickson Torio[1], Hirotaka Ito[1], Junfeng Liu[1], Naoyuki Shono[1], Michal O. Nowicki[1], Daniel Triggs[1], Patrick Halloran[1], Raziye Piranlioglu[1], Himanshu Soni[1], Brittany Stopa[1], Wenya Linda Bi[1], Pierpaolo Peruzzi[1], Ethan Chen[1], Seth W. Malinowski[2], Michael C. Prabhu[2], Yu Zeng[2], Anne Carlisle[6], Scott J. Rodig[2,6], Patrick Y. Wen[7], Eudocia Quant Lee[7], Lakshmi Nayak, M.D.[7], Ugonma Chukwueke, M.D.[7], L. Nicolas Gonzalez Castro[7,8], Sidney D. Dumont[9,10], Tracy Batchelor.[8], Kara Kittelberger[11], Ekaterina Tikhonova[12], Natalia Miheecheva[12], Dmitry Tabakov[12], Nara Shin[12], Alisa Gorbacheva[12], Artemy Shumskiy[12], Felix Frenkel[12], Estuardo Aguilar-Cordova[13], Laura K. Aguilar[13], David Krisky[13], James Wechuck[13], Andrea Manzanera[13], Chris Matheny[13], Paul P. Tak[13], Francesca Barone[13], Daniel Kovarsky[14], Itay Tirosh[14], Mario L. Suvà[9,10], Kai W. Wucherpfennig[15], Keith Ligon[2], David A. Reardon[7], E. Antonio Chiocca[1]*.

Affiliations:
[1] Harvey Cushing Neuro-oncology Laboratories, Department of Neurosurgery, Hale Building for Transformative Medicine, 4th and 8th floor, Brigham and Women's Hospital; 60 Fenwood Street, Boston, MA 02116.
[2] Department of Pathology, Brigham and Women's Hospital, 75 Francis St, Boston, MA 02115 and Department of Pathology, Dana-Farber Cancer Institute, 450 Brookline Avenue, Boston, MA 02115.
[3] Department of Biostatistics, Dana Farber Cancer Institute; Boston, MA 02116
[4] Center for Biostatistics, Department of Biomedical Informatics, The Ohio State University; Columbus, OH
[5] James Comprehensive Cancer Center, The Ohio State University; Columbus, OH
[6] Center for Immuno-Oncology, Dana-Farber Cancer Institute
[7] Center for Neuro-oncology, Dana Farber Cancer Institute; Boston, MA 02116
[8] Department of Neurology, Brigham and Women's Hospital; 60 Fenwood Street, Boston, MA 02116.
[9] Department of Pathology and Center for Cancer Research, Massachusetts General Hospital, Boston, MA 02114
[10] Broad Institute of MIT and Harvard, Cambridge, MA 02142, USA [11]ClearPoint Neuro, Inc., 120 S. Sierra Ave, Solana Beach, CA 90275.
[12] BostonGene, Corp., 95 Sawyer Rd., Suite 500, Waltham, MA 02453.
[13] Candel Therapeutics, Inc, Needham, MA 02494
[14] Department of Molecular Cell Biology, Weizmann Institute of Medical Sciences, Tel Aviv, Israel.
[15] Department of Cancer Immunology and Virology, Dana Farber Cancer Institute; Boston, MA 02116

*To whom correspondence should be addressed at: Department of Neurosurgery, Hale Building for Transformative Medicine, 4th floor, Brigham and Women's Hospital, 60 Fenwood Street, Boston, MA 02116. Email: eachiocca@bwh.harvard.edu
†This author should be considered as a first co-author.

This PDF file includes:

Links to Supplementary Tables 1 - 3
Link to Supplementary Video 1
Methods
End Notes
Supplementary Figure 1
Supplementary References 50-72 (continued from main text citations[1–49])

Supplementary Tables

Supplementary Table 1: Patient treatment histories (related to Sub-heading, *HSV1 serology predicts efficacy*).

Please see attached excel table or link:

https://www.dropbox.com/scl/fi/hij2mzjpqzi3axgsjx6yz/Table-S1_Patient-Treatment-Histories.xlsx?rlkey=1jnvyov2a9vf3xv3qvqm04mmq&dl=0

Supplementary Table 2a-c: Patient Tissue Summary and Pathology quantifications (related to Sub-Headings, *CAN-3110 increases T cells in tumors*, and *OV persistence links to seronegativity*)

Please see attached excel table or link:

https://www.dropbox.com/scl/fi/twodv1iek7b3reuvzqhug/Table-S2_Patient_Tissue_Summary_and_Pathology_Quantifications.xlsx?rlkey=t4mvtf418uem5l4jy5ukf0umv&dl=0

Supplementary Table 3: TCR sequences that were statistically changed in TILs following CAN3110 (related to Sub-heading, C*hanges in T cell repertoire.*).

Please see attached excel table or link:

https://www.dropbox.com/scl/fi/plq2oli2dlttyvzxthi3x/Table-S3_TCR-sequences-that-were-statistically-changed-in-TILs-following-CAN3110.xlsx?rlkey=2kjedntu75u36xm1rziwicrim&dl=0

Supplementary Video

Supplementary Video 1: Subject 021 experience with CAN-3110. Subject 021 underwent standard of care surgery and chemoradiation for her GBM, but then recurred. After a second surgery, she recurred rapidly. She then underwent intraoperative MRI-guided stereotactic injection of CAN-3110. An MRI 90 days later, appeared to show recurrence again and she underwent a third resection. This resection showed significant novel infiltration with CD8+ T cells. After this, she remained tumor-free by MRI for almost 2 years with adjuvant pembrolizumab infusions. She succumbed to a motor vehicle accident where she was the passenger. The video features pictures and videos of Susan as well as interviews with her daughter.

https://www.dropbox.com/s/pdvp2rsutdfysel/Supplemental%20video%201%20downscaled.mp4?dl=0

Methods

Clinical Protocol (eligibility criteria). This phase 1 clinical trial was reviewed and approved by NIH RAC Office of Biotechnology Affairs (NIH no 1104-1100) and the IRB from the DFCI (no 16-557). The IND Sponsor was Dr. Chiocca (IND 16380). Eligibility was restricted to subjects with pathologically confirmed high-grade gliomas that failed prior surgery, radiation with temozolomide and other clinical trials or chemotherapies. MRI evidence of possible recurrent glioma had to be available. Subjects had to be judged appropriate surgical candidates by their attending neurosurgeon. The following were the eligibility criteria:

1. At the time of surgery, frozen biopsy confirmation of high grade or malignant glioma by neuropathologist. Biopsy confirmation of glioma or infiltrative glioma at time of surgery was acceptable, provided that the subject had prior pathology confirmation of high-grade glioma. If a subject had a previous diagnosis of low grade glioma, then the biopsy had to show high grade glioma.
2. Participants must have had prior diagnosis of glioma (astrocytoma, malignant astrocytoma, oligodendroglioma, anaplastic oligodendroglioma, mixed oligo-astrocytoma), exclusive of ependymoma, ganglioglioma, pylocytic/pylomyxoid astrocytoma as confirmed by a neuropathologist or by a previous local pathology report.
3. Prior history of external beam radiotherapy $\geq$ 5,000 cGy delivered to the tumor at least 4 weeks prior to OHRS registration. Participants over the age of 70 with prior history of hypofractionated external beam radiotherapy were also accommodated, in accordance with NCCN guidelines.
4. For participants with diagnosis of oligodendroglioma or anaplastic oligodendroglioma, prior history of external beam radiotherapy < 5,000 cGy or no history of radiation were acceptable.
5. Prior history of temozolomide chemotherapy provided concurrent to external beam radiotherapy and after as per current standard of care. However, temozolomide was not required to have been provided concomitantly or after radiation if the patient had unmethylated MGMT promoter or if the patient initially was diagnosed with a low grade glioma. At least 4 weeks must have passed from the last dose of temozolomide and first dose of CAN-3110 (e.g., rQNestin34.5v.2).
6. For participants with diagnosis of oliogodendroglioma or anaplastic oligodendroglioma, chemotherapy was administered before, during, or after radiation or not at all.
7. If participant was treated with bevacizumab, at least 4 weeks had to elapse before treatment with CAN-3110.
8. Recurrent lesion had to be $\geq$ 1.0 cm in diameter as determined by MRI.
9. Normal hematological, renal and liver function as defined below:

– Leukocytes	≥3,000/mcL
– Absolute lymphocyte count	> 500/ mcL
– Absolute neutrophil count	≥1,500/mcL
– Platelets	≥100,000/mcL
– PT or PTT	<1.5 x institutional upper limit
– Hemoglobin	>10.0 g/dL
– Total serum bilirubin	within normal institutional limits
– AST(SGOT)/ALT(SGPT)	≤2.5 × institutional upper limit of normal
– Serum creatinine	≤ 1.5 mg/dL
	OR
– Creatinine clearance	≥60 mL/min/1.73 m^2 for participants with creatinine levels above institutional normal.

10. Karnofsky Performance Score $\geq$ 70.
11. Age $\geq$ 18 years.
12. Ability to understand and the willingness to sign a written informed consent document.
13. The effects of CAN-3110 on the developing human fetus reamin unknown. For this reason, women of child-bearing potential and men had to agree to use adequate contraception (hormonal or barrier method of birth control; abstinence) prior to study entry and for the duration of study participation including 3 months following the study.

If a woman became pregnant or suspected pregnancy while she or her partner were participating in the study, they should inform the treating physician immediately. Men treated or enrolled on this protocol had to also agree to use adequate contraception prior to the study and for the duration of study participation including 3 months following the study. Women of child-bearing potential had to have a negative serum pregnancy test within 48 hours of study registration.

14. Steroid regimen stable or decreasing for at least 7 days prior to inoculation.
15. Ability to undergo MRI scanning with contrast.
16. Subjects with any recurrence (first, second, third, etc recurrence) will be able to be enrolled.

The following were exclusion criteria:

1. Participants with significant renal or liver disease.
2. Participants with progressive systemic malignancy.
3. Known chronic infections with HIV, hepatitis B or C; participants with a history of resolved Hepatitis A may be included in the trial.
4. Participants with active viral, bacterial or fungal infection requiring concurrent antiviral or antibiotics.
5. Subjects with active HSV-1 infection on current valacyclovir, acyclovir or ganciclovir therapy must be off treatment with any of these agents at least 7 days prior to surgery.
6. Active, known, or suspected immunosuppressive disorders, such as acquired or congenital immune deficiency syndromes and autoimmune diseases.
7. Unacceptable anesthesia risk.
8. Serious cardiopulmonary medical condition.
9. Pregnant or lactating females.
10. Recurrent glioma where injection of the biologic agent would require access and/or considerable spillage into the ventricular system.
11. Prior participation in another protocol using an investigational agent within 5 half-lives or 4 weeks of the investigational agent, whichever was shorter.
12. Known HIV seropositivity.
13. Concurrent therapy with drugs active against HSV (acyclovir, valaciclovir, penciclovir, famciclovir, ganciclovir, foscarnet, cidofovir). Participants must be off treatment with these agents for at least 7 days prior to surgery.
14. Active oral or genital herpes lesions.
15. Participants who have had chemotherapy or radiotherapy within 4 weeks prior to entering the study or those who have not recovered from adverse events due to agents administered more than 4 weeks earlier.
16. Participants who are receiving any other investigational agents. Previous CAN-3110 participants may be re-enrolled if they completed at least the day 56 assessment without DLT.
17. Uncontrolled intercurrent illness including, but not limited to, ongoing or active infection, symptomatic congestive heart failure, unstable angina pectoris, cardiac arrhythmia, or psychiatric illness/social situations that would limit compliance with study requirements.
18. Participants with tumor ≤ 1 cm proximity to the ventricles were allowed to enroll. However, the study agent (CAN-3110) could not be injected in any area that was within 1 cm of the ventricle regardless of where the tumor was located.

Study design/population (related to sub-heading "Safety of CAN-3110 in rHGG/rGBM patients" in main text and Tables 1a, b). The oHSV, CAN-3110 (former designation, rQNestin34.5v.2) was tested preclinically in glioma xenograft and naïve murine brain models establishing a non-lethal dose of 10^3 plaque-forming units (PFUs), with neurotoxicity at higher doses[3]. Based on preclinical data and with FDA guidance, an open label, single institution, phase 1 trial in humans with real-time frozen section pathologic confirmation of high-grade glioma was conducted to determine the safety of CAN-3110, starting with a single intra-tumoral injection of 10^6

PFUs and dose-escalating (3+3 design) by half-log increments up to 10^{10} PFUs (cohorts 1-9). The starting human dose was selected as a dose that had shown no lethality in mice (at its bioequivalent mouse dose)[3]. An expansion cohort (cohort 10) was added at a dose of 10^9 PFUs injected into up to 5 sites in a single tumor **(Extended Data Figure 1a)**. To ensure injection into gadolinium-enhancing tumor and avoid reflux, injections were carried out in an intraoperative MRI using a stepped needle design **(Extended Data Figure 1b)** over a period of 5 minutes**.** After injection, an intraoperative MRI was obtained visualizing an area of darkening on the T1 weighted MRI sequences corresponding to the injectate **(Extended Data Figure 1c).** Thirty patients were treated from September 2017 until February 2020 in 9 dose cohorts up to 10^{10} PFUs in 1 ml volumes **(Extended Data Table 1a).** An additional 12 subjects (one of these was a repeat treatment as discussed above) were treated from June 2020 until January 2021 in an expansion cohort (cohort 10) where the enhancing tumor was injected in up to 5 areas based on tumor diameter with 10^9 PFUs diluted into 2-5 ml of buffer (1 ml per injection site) **(Extended Data Table 1b)**. These results thus showed that CAN-3110: **a-** could be injected into rHGG/rGBM with MRI evidence of injectate within tumor with minimal if any reflux, **b-** single timepoint injection of doses up to 10^{10} pfus were technically feasible, and **c-** single timepoint injection into multiple (up to 5) regions of a rHGG/rGBM were also feasible.

Detailed Neurosurgical Procedure.
A dose of 1 mL of CAN-3110 diluted to the required titer was used for Cohorts 1 to 9. For Cohort 10, the injected volume was based on the estimated size of the tumor and feasibility according to the table below and can range from 2 to 5 ml (2 to 5 injections of one milliliter) targeting a total viral dose $1x10^9$ plaque forming units, as per the table below.

**Volume of virus injection for Cohort 10**	
Maximum tumor dimension	Total volume of CAN-3110 vector
1 cm – 2 cm	Up to 2 ml (2 injections of 1ml)
2.001 cm – 3 cm	Up to 3 ml (3 injections of 1ml)
3.001 cm – 4 cm	Up to 4 ml (4 injections of 1ml)
4.001 cm or greater	Up to 5 ml (5 injections of 1ml)

To prepare the dose, the vial was thawed on cold packs in the Brigham Research Pharmacy and dilution to required titer performed in a biosafety hood by the research pharmacist. Sterile technique and Biosafety Level 2 precautions (gown, gloves, mask) were rigorously followed while preparing the dose. After initial thawing and dilution, the dose was injected within 5 hours.

Each subject underwent general anesthesia and then surgery was performed in the intraoperative MRI. After standard MRI-guided biopsy and confirmation of tumor, the dose of CAN-3110 was administered on day 0 (see schema). There were 9 cohorts in the dose escalation scheme from 10^6 PFU up to 10^{10} PFU by ½ log increments followed by a 10th cohort where the safety of multiple tumor injections will be tested (two to five injections of one milliliter per injection) with a total virus dose of like that of cohort 7, $1x10^9$ plaque forming units. The injection(s) occured in the intraoperative MRI operating room to confirm needle placement in tumor. The time of the inoculation was 5 minutes (approximately 100 microliters every 30 seconds) per one milliliter dose using the SmartFlow Cannula® (NGS-NC-01, 16 ga, 0.008" I.D. x 4 ft., ClearPoint Neuro, Inc.). After injection the needle was left in place for 1 minute before being slowly withdrawn over the following minute. Intraoperative MRIs were obtained during the procedure to confirm biopsy region and then to confirm needle placement including a final MRI after injection to confirm region of inoculation. After surgery, the subject was awoken from anesthesia. Most subjects left on postoperative day 1 or 2. A postoperative standard MRI was obtained within 48 hours of surgery.

CAN-3110 safety (related to sub-heading ""Safety of CAN-3110 in rHGG/rGBM patients" in main text and Table 1a, b). In **Extended Data Table 2**, all treatment phase AEs (grade 1 or 2) reported to date possibly, probably or definitely related to CAN-3110 are shown.

Clinical protocol: definition of adverse event (AE), serious adverse event (SAE), dose limiting toxicity (DLT), and maximum tolerated dose (MTD).

An adverse event (AE) was any undesirable sign, symptom or medical condition or experience that developed or worsened in severity after starting the first dose of study treatment or any procedure specified in the protocol, even if the event was not considered to be related to the study. Abnormal laboratory values or diagnostic test results constituted adverse events only if they are considered clinically significant. Clinically significant values had to meet one or more of the following:

- Required change in study medication (e.g., dose modification, interruption or permanent discontinuation)
- Required additional follow up (ie repeat labs, additional tests, etc)
- Resulted in clinical signs &/or symptoms
- Required change in concomitant therapy (e.g., addition of, interruption of, discontinuation of, or any other change in a concomitant medication, therapy or treatment)

The descriptions and grading scales found in the revised NCI Common Terminology Criteria for Adverse Events (CTCAE) version 4.0 was initially utilized for AE reporting and then version 5.0 was utilized as the trial progressed. Grading of AE referred to the severity of the AE. The CTCAE displays Grades 1 through 5 with unique clinical descriptions of severity for each AE based on this general guideline:

- **Grade 1** Mild; asymptomatic or mild symptoms; clinical or diagnostic observations only; intervention not indicated.
- **Grade 2** Moderate; minimal, local or noninvasive intervention indicated; limiting age-appropriate instrumental activities of daily living (ADL).
- **Grade 3** Severe or medically significant but not immediately life-threatening; hospitalization or prolongation of hospitalization indicated; disabling; limiting self-care ADL.
- **Grade 4** Life-threatening consequences; urgent intervention indicated.
- **Grade 5** Death related to AE.
-

Attribution of AE was done by the study team and consisted of:

- Definite – The AE *is clearly related* to the study treatment.
- Probable – The AE *is likely related* to the study treatment.
- Possible – The AE *may be related* to the study treatment.
- Unlikely – The AE *is doubtfully related* to the study treatment.
- Unrelated – The AE *is clearly NOT related* to the study treatment.
-

Serious Adverse Event (SAE) meant any event temporally associated with the subject's participation in research that met any of the following criteria:

1) Death
2) A life-threatening adverse event
3) An adverse event that results in inpatient hospitalization or prolongation of existing hospitalization for ≥ 24 hours. There were exclusions on the definition of hospitalization: Rehabilitation facilities; Hospice facilities; Respite care (e.g., caregiver relief); Skilled nursing facilities; Nursing homes; Routine

emergency room admissions; Same day surgeries (as outpatient/same day/ambulatory procedures); Admission for treatment of a preexisting condition not associated with the development of a new adverse event or with a worsening of the preexisting condition (e.g., for work-up of persistent pre-treatment lab abnormality); Social admission (e.g., subject has no place to sleep); Administrative admission (e.g., for yearly physical exam); Protocol-specified admission during a study (e.g., for a procedure required by the study protocol); Optional admission not associated with a precipitating clinical adverse event (e.g., for elective cosmetic surgery); Pre-planned treatments or surgical procedures should be noted in the baseline documentation for the entire protocol and/or for the individual subject; Admission exclusively for the administration of blood products. In addition, diagnostic and therapeutic non-invasive and invasive procedures, such as surgery, were not reported as adverse events.

4) A persistent or significant incapacity or substantial disruption of the ability to conduct normal life functions.
5) A congenital anomaly/birth defect.
6) Important Medical Events (IME) that may not result in death, be life threatening, or require hospitalization may be considered serious when, based upon medical judgment, they may jeopardize the patient or subject and may require medical or surgical intervention to prevent one of the outcomes listed in this definition. (FDA, 21 CFR 312.32; ICH E2A and ICH E6).

Based on the unique features of CAN-3110, additional toxicities were prospectively evaluated for:

a) Herpes encephalitis/meningitis. This could be easily diagnosed and a clinical concern for the occurrence of herpes encephalitis/meningitis would prompt the consideration for intravenous administration of anti-herpetic medications. CAN-3110 is sensitive to acyclovir/valacyclovir/ganciclovir administration. Central nervous system herpes infection can manifest as: severe meningeal irritation, uncontrolled seizure, obtundation/coma, high grade fevers. MR/CT may show diffuse edema and/or hemorrhagic foci in frontal and/or temporal lobes and/or diffuse enhancement of meninges. Intravenous acyclovir would be started at 10 milligrams/kilogram (mg/kg) infused intravenously, at a constant rate over 1 hour, every 8 hours for 10 days. The maximum dose is 20 mg/kg every 8 hours (Prod Info ZOVIRAX(R) IV injection, 2003). In the event of any early symptoms of encephalitis such as confusion, etc, viral DNA levels in the CSF will be performed in addition to the MRI.

b) Spread of the oHSV. It is possible that some of the injected CAN-3110 could spread to other parts of the patient’s body with unknown effects. Since the agent was rendered fairly replication-selective for tumors and it is also derived from a HSV1 strain (F strain) that is sensitive to complement inactivation, viremias were likely to be transient and may manifest themselves with flu-like symptoms.

c) Changes in the body's egg or sperm. It is theoretically possible that the egg or sperm may be affected. This would have unknown effects on future births or development of children. In this population, it is unlikely that this was an issue. However, it was still recommended that the subject use birth control for the duration of the study. A pregnancy test was performed on women of childbearing years prior to study entry. Pregnancy tests done after drug administration were done as clinically indicated.

d) It is possible that CAN-3110 could reactivate an existing herpes that may be latent (dormant) in the subject. There is no evidence from animal studies, or prior human studies in treatment of brain tumor with herpes simplex viruses to suggest that vector inoculation could lead to reactivation of latent HSV from ganglia or brain, but this possibility must be considered. It is also possible that CAN-3110 could mutate only through recombination with a wild type virus. Although this has not occurred in human studies to date, recombination of CAN-3110 with a wild-type HSV1 is likely to generate an HSV1 that is more attenuated than the wild-type HSV1, since all the changes in CAN-3110 make it more selective for tumors.

The maximum tolerated dose (MTD) was defined as the dose one half-log order less than the dose level at which one-third of the subjects have a dose limiting toxicity (DLT) of grade 3 or grade 4 (defined below) related to the administration of CAN-3110. Cohorts of three subjects were administered with the CAN-3110 dose escalated by half-log increments at each dose level until a DLT is reached. If the MTD was not reached, the phase I dose was the highest dose reached (HTD = highest treatment dose).

The first subject in each cohort was observed for at least 14 days following injection with CAN-3110 prior to the next subject being enrolled into the study protocol. If there was no DLT, then the second and third subject were accrued at the same dose. Subjects were only enrolled into the next dose level if 1/3 DLT was not reached based on CTCAE v.4.

A DLT consisted of:

- Any Grade 4 or 5 toxicity on the Common Terminology Criteria for Adverse Events v4.0 (CTCAE) attributed to CAN-3110, except for Grade 4 lymphocyte, neutrophil, white blood count decrease on the investigation category of CTCAE v4.0.
- Any grade 3 or greater non hematologic toxicity as defined by NCI CTCAE v.4, and considered as being possibly, probably or likely related to study product or study product that was not reversible within 2 weeks or any life-threatening event, or treatment-related death was considered a DLT. Any grade 2 or higher serious autoimmune toxicities particularly those affecting vital organs (e.g. cardiac, renal, CNS) were considered a DLT if it occurs within 2 weeks with or without treatment.
- Grade 3 or 4 myelosuppression defined as lymphopenia, neutropenia, leukopenia that has resolved within 21 days of study agent administration was not considered a DLT.
- Grade 3 or greater hematuria was considered a DLT.

Additional exceptions to Dose Limiting Criteria (DLT) were:

- **Seizures:** Subjects may have pre-existing seizures or be susceptible to new seizures as a result of the underlying disease process. Seizures were considered DLT unless they have increased in frequency and are not attributed to another recognized cause of increasing seizure frequency, such as subtherapeutic anti-convulsant levels or biopsy-proven tumor progression. Seizures defined as Grade 3 or 4 toxicities under NCI CTCAE, should still be reported as such in the proposed trial even if not considered DLT.
- **New neurologic deficits:** Subjects may develop new neurologic deficits as a result of tumor invasion. A new neurologic deficit which resolved within 2 weeks after initiation of medical therapy was not considered a DLT. Similarly, a late (i.e., 4 weeks or longer after completion of infusion) adverse event that is thought to be drug- or procedure-related and resolves within 2 weeks after initiation of medical therapy, was not considered a DLT. New neurological symptoms were not a DLT if they could be ascribed to tumor progression [e.g., documented with histopathologic analyses of biopsy tissue or responded to treatment (e.g., oral steroids within 2 weeks)].
- **Syndrome of Inappropriate Antidiuretic Hormone (SIADH):** Subjects may develop SIADH due to the underlying disease process. SIADH was not considered DLT unless it is refractory to medical management. SIADH defined as a DLT under NCI CTCAE should still continue to be reported as such in the proposed trial even if not considered DLT.
- **Muscle Weakness and Weight Gain:** Subjects may develop muscle weakness or weight gain due to steroids instituted to treat disease progression. Muscle weakness may be defined as Grade 3 or Grade 4 toxicity and weight gain of 20% or more may be defined as Grade 3 toxicity under NCI CTC and should be reported as such in this study. However, muscle weakness or weight gain were not considered DLT if the subject had required steroids greater than physiologic doses in the interval between the immunization and the development of the muscle weakness or weight gain.

- **<u>Tumor Progression</u>:** Subjects may have an increase in pre-existing neurologic deficits or have an onset of new neurologic deficits due to tumor progression. Although such neurologic deficits may be defined as DLTs under NCI CTCAE and should be reported as such in this study, neurologic deficit were not considered a DLT if unequivocal tumor progression can be documented radiographically or histologically, and the deficit is attributable to the tumor progression.

Management of Toxicities:
If a Grade 3 or 4 toxicity were observed, the subject was monitored until the toxicity improved to Grade 1 or better. If the toxicity resolved within 2 weeks, either with or without treatment, this toxicity was not considered dose-limiting. If a new Grade 3 or 4 toxicity was seen again in the same subject after initial resolution within 2 weeks of the initial infusion, a DLT would be declared. If Grade 2 or higher serious autoimmune toxicity is observed, the subject was monitored until the toxicity improved to Grade 1 or better. If resolution to Grade 1 occurred within 4 weeks with or without treatment, this toxicity was not considered dose limiting.
Dose escalation proceeded within each cohort according to the following scheme:

Number of Participants with DLT at a Given Dose Level	Escalation Decision Rule
0 out of 3	Enter 3 participants at the next dose level.
<u>></u>2	Dose escalation will be stopped. This dose level will be declared the maximally administered dose (highest dose administered). Three (3) additional participants will be entered at the next lowest dose level if only 3 participants were treated previously at that dose.
1 out of 3	Enter at least 3 more participants at this dose level.<br>• If 0 of these 3 participants experience DLT, proceed to the next dose level.<br>• If 1 or more of this group suffer DLT, then dose escalation is stopped, and this dose is declared the maximally administered dose. Three (3) additional participants will be entered at the next lowest dose level if only 3 participants were treated previously at that dose.
≤1 out of 6 at highest dose level below the maximally administered dose	This is generally the recommended phase 2 dose. At least 6 participants must be entered at the recommended phase 2 dose.

Prior to achieving MTD, the study would have been paused in the following instances:

- Death
- 2 grade 4 events
- Encephalitis

CAN-3110 efficacy (related to sub-heading "HSV1 serology predicts efficacy*" *in main text and Figure 1). Progression-free survival curves are shown in **Extended Data Figures 3a-b.** After CAN-3110, if subjects developed neurologic decline due to edema, treatments with steroids and/or low dose bevacizumab (5mg/kg) were

instituted **(Extended Data Figures 3c, d, Supplemental Table 1)**. The latter usually resulted in rapid resolution of symptoms and significant reduction in enhancement. If progression and/or pseudo-progression[50] was suspected or proven by tissue analysis from biopsy/re-resection, a variety of additional treatments were instituted as illustrated in the swimmer's plots of **Extended Data Figures 3c,d** for cohorts 1-9 and 10, respectively. These results show that the clinical course for these rHGG/rGBM subjects was relatively typical for this patient population.

Clinical Protocol: response assessment

The original clinical trial protocol used serial MRI scans obtained approximately every 8 weeks**.** A separate publication is in preparation using the original MRI-based assessments of response to determine outcomes of the trial. The MRI scan done within 72 hours of surgery was the baseline scan for tumor response assessments. Tumor response was evaluated radiographically using MRI scans to determine tumor response and to assess the time of disease progression. Response and progression were evaluated in this study using modified RANO criteria. Measurable disease was defined as bi-dimensionally contrast-enhancing lesions with clearly defined margins by CT or MRI scan, with two perpendicular diameters of at least 10 mm, visible on two or more axial slices that are preferably, at most, 5 mm apart with 0-mm skip. Enhancement around cysts or surgical cavities is in general considered non-measurable unless there is a nodular component measuring ≥ 10 mm in diameter. The cyst or surgical cavity were not measured in determining response. Non-measurable disease was defined as either uni-dimensionally measurable lesion, masses with margins not clearly defined, or lesions with maximal perpendicular diameter < 1 cm. All measurements were taken and recorded in metric notation using a ruler, calipers, or a digital measurement tool.

Response criteria were:

Complete response (CR): All of the following criteria must be met:

- Complete disappearance of all enhancing measurable and non-measurable disease sustained for at least 4 weeks. In the absence of a confirming scan 4 weeks later, this scan will be considered only stable disease.
- No new lesions.
- All measurable and non-measurable lesions must be assessed using the same techniques as baseline.
- Participants must be on no steroids or on physiologic replacement doses only.
- Stable or improved non-enhancing (T2/FLAIR) lesions.
- Stable or improved clinically, for clinical signs and symptoms present at baseline and recorded to be disease related

NOTE: Participants with non-measurable disease cannot have a complete response. The best response possible is stable disease.

Partial response (PR): All of the following criteria must be met:

- Greater than or equal to 50% decrease compared to baseline in the sum of products of perpendicular diameters of all measurable enhancing lesions sustained for at least 4 weeks.
- No progression of non-measurable disease
- No new lesions.
- All measurable and non-measurable lesions must be assessed using the same techniques as baseline.
- The steroid dose at the time of the scan evaluation should be no greater than the dose at time of baseline scan.
- Stable or improved non-enhancing (T2/FLAIR) lesions on same or lower dose of corticosteroids compared to baseline scan.
- Stable or improved, for clinical signs and symptoms present at baseline and recorded to be disease related

clinically.

NOTE: Participants with non-measurable disease cannot have a partial response. The best response possible is stable disease.

Stable Disease (SD): All of the following criteria must be met:

- Does not qualify for CR, PR, or progression.
- All measurable and non-measurable sites must be assessed using the same techniques as baseline.
- Stable clinically.

Progressive Disease (PD): All of the following criteria must be met:

- 25% increase in sum of the products of perpendicular diameters of enhancing lesions (over best response or baseline if no decrease) on stable or increasing doses of corticosteroids *and/or one or more of the following:*
- Any new measurable enhancing lesion
- Clear clinical deterioration not attributable to other causes apart from the tumor (e.g. seizures, medication side effects, complications of therapy, cerebrovascular events, infection, etc.). The definition of clinical deterioration is left to the discretion of the investigator but may include a decline in the Karnofsky Performance Score (KPS) from 100 or 90 to 70 or less, a decline in KPS of at least 20 from 80 or less, or a decline in KPS from any baseline to 50 or less, for at least 7 days, unless attributable to comorbid events or changes in concurrently administered medications.
- Failure to return for evaluation due to death or deteriorating condition

NOTE: Participants with non-measurable disease cannot have a complete response. The best response possible is stable disease.

Brain tumor tissue collection and allocation

Prior to CAN-3110 (rQNestin34.5v2 oHSV) injection, recurrence of high-grade glioma was confirmed by intraoperative evaluation of surgical biopsy material, including frozen sections and smear preparations stained with hematoxylin and eosin. The remainder of the frozen tissue was then transferred to 10% neutral buffered formalin. Additional core needle biopsies were also transferred to 10% neutral buffered formalin for routine histopathology, neural stem cell media for patient derived model creation, frozen for molecular studies, or fresh for cell sorting. Post-treatment surgical biopsy or resection specimens underwent intraoperative evaluation at the discretion of the neurosurgeon, and tissue was allocated as per the pre-injection biopsies. Post-mortem brain evaluations were performed following consent by the next-of-kin or healthcare proxy per the applicable state laws. Autopsies performed at BWH included freezing representative sections of tumor from a single coronal slice and grossly uninvolved brain tissue, and the remainder of the brain tissue was fixed formalin for two weeks prior to additional sectioning and extensive tumor sampling (30-40 cassettes per case). Subjects who underwent brain removal at other institutions had brains fixed in formalin for two weeks prior to shipping to BWH for examination as above.

Immunohistochemistry (related to sub-heading "CAN-3110 increases T cells" in main text and Figure 2). Routine and trial-based post-CAN3110 neuro-oncological care included serial clinical, imaging and laboratory follow-up, daily in the post-operative period and then every 8 weeks **(Extended Data Figures 3c, d)**. When necessary and as part of standard neuro-oncologic care, a repeat craniotomy to determine pathologic evidence of progression or inflammatory pseudo-progression was carried out at various timepoints after CAN-3110 injection (range= 7 – 801 days) **(Supplementary Tables 2a-c)**. Therefore, these repeat craniotomies resulted in the collection of 23/42 separate tumors after CAN-3110 (in 41 subjects since subject 042 also underwent a second timepoint injection with CAN-3110 as subject 054 with a subsequent re-resection). Further, 7 autopsies were

performed in previously treated subjects and 4 autopsies were also performed in patients that had also previously undergone a resection post-CAN-3110 (for a total of 11 autopsies). Therefore, a total of 30 unique paired tumors were available to compare pre- and post-CAN-3110 changes. We also had tumors from multiple resections (n = 4, subject 042 and subject 054 who are the same patient but underwent 2 separate timepoint injections followed by re-resection are counted separately) or from resection and autopsy (n = 4) after CAN-3110. One subject (048) underwent a resection after CAN-3110 too late to include in this analysis. Therefore only 29 pre- and post-CAN3110 tumors are reported. These post-injection tumors when matched with their pre-injection tumors provided the paired samples analyzed in **Figures 2-5, Extended data Figures 5-13,** and **Supplementary Tables 2, 3**

Histopathology, immunohistochemistry, and digital quantification

Formalin-fixed tissue from surgical and autopsy cases was processed and paraffin-embedded, with H&E sections prepared from all blocks. Tumor percentage and necrosis were estimated by visual inspection by a board-certified neuropathologist. Post-treatment specimens were stained with HSV-1 polyclonal antibody (Dako B0114). One block from each pre-treatment biopsy and post-treatment surgical/autopsy case (with positive HSV-1 IHC and/or the greatest amount of inflammation observed on H&E), was stained for CD4 (Dako 4B12), CD8 (Dako 144B), CD20 (Dako L26), Nestin (Cell Signaling Technologies 10C2), and Nectin-1/CD111 (Santa Cruz Biotech CK6). Slides were scanned using the Hamamatsu NanoZoomer S210 at 40x magnification. CD4+ and CD8+ T cells, CD20+ B cells, and immunonegative cells were quantified using HALO Image Analysis Software (PerkinElmer). Three square regions of interest (approximately 160,000 um^2 each) were averaged for each case in areas of non-necrotic tumor and in areas of tumor with high levels of inflammation adjacent to necrosis if present. The quantities of IHC positive cells were normalized by tissue area (mm^2). Graphs were created in Prism with Wilcoxon signed rank test. Nestin and Nectin-1 IHC slides were interpreted as positive or negative. Antibody dilutions were as follows:

**Target**	**Company**	**Clone**	**Antibody Dilution**
CD4	Dako	4B12	1:80
CD8	Dako	144B	1:100
CD20	Dako	L26	1:250
HSV-1	Dako	Polyclonal	1:1000
Nestin	Cell Signaling Technologies	10C2	1:800
Nectin-1/CD111	Santa Cruz Biotech	CK6	1:100

All chromogenic immunohistochemistry utilized commercially available antibodies optimized for staining formalin-fixed paraffin-embedded tissue sections, with staining performed in a CAP certified laboratory.

Multiplex Immunofluorescence

Multiplex immunofluorescence staining was performed as previously described[51]. In brief, BOND RX fully automated stainers (Leica Biosystems) were used to stain for SOX2 (tumor cells), CD68 and CD163 (macrophages/microglia), and PD-L1. Image acquisition was performed using the Mantra multispectral imaging platform (Vectra 3, PerkinElmer). Representative regions of interest were chosen by the pathologist, and multiple fields of view were acquired at 20× resolution as multispectral images. Areas of tumor adjacent to and distant from the area of HSV positive necrosis were analyzed separately in the post-treatment samples. Cell identification was performed using supervised machine learning algorithms within Inform 2.3 (PerkinElmer). Thresholds for 'positive' staining and the accuracy of phenotypic algorithms were optimized and confirmed by the pathologist (S.J.R.) for each case. Quantification of this project was done using Akoya Inform Automated Image Analysis Software version 2.4.8. Antibody dilutions were as follows:

Target	Company	Clone	Antibody Dilution	Opal Fluorophore	Fluor Dilution
Sox2	Cell Signaling Technology	D6D9	1:250	620	1:200
CD68	Dako	PG-M1	1:1500	650	1:100
CD163	Novocastra	10D6	1:1500	570	1:225
PD-L1	Cell Signaling Technology	E1L3N	1:200	520	1:100

All of these antibodies are commonly used. Each antibody was first optimized by standard IHC to confirm fidelity of the staining, then adapted to single-immunofluorescence staining before combining antibodies together into a multiplex immunofluorescence panel. In single-immunofluorescence, repeated rounds of optimization include testing different antigen retrieval conditions, diluents and a wide range of antibody concentrations. In multiplex, different panel conditions are tested to ensure high signal to noise for each individual marker, while eliminating bleedthrough, crosstalk between channels, and nonspecific staining.

Tumor Genotyping

Surgical specimens from 34 trial patients were analyzed by targeted next-generation sequencing via DFCI Oncopanel (n=33)[52] or MSK-IMPACT (n=1)[53]. These validated measures identify genomic alterations including single-nucleotide variants, insertions and deletions, copy number alterations, and structural variants in the exons and selected introns of 447 (Oncopanel) and 410 (MSK-IMPACT) genes, including genes relevant to the diagnosis and subclassification of HGG. Sequencing data from these assays, as well as clinical information regarding age, trial cohort, diagnosis at time of injection, and MGMT promoter methylation status, was used to generate an Oncoprint genomic profile (Fig.S3) using R 4.2.1, RStudio 2022.07.2+576, and the Oncoprint function of the ComplexHeatmap 2.12.1 package. Results of sequencing for each brain tumor were reviewed by a board-certified neuropathologist and incorporated with the histological findings into a final integrated diagnosis.

Data plotting and statistical analyses

Data plotting and statistical analyses were performed using R (version 4.1.0)[54] and RStudio (version 2022.2.3.492)[55] along with the following packages: *openxlsx* (version 4.2.5)[56]; *ggplot2* (version 2_3.3.5)[57]; *tidyverse* (version 1.3.1)[58]; *rstatix* (version 0.7.0)[59]; *ggpubr* (version 0.4.0)[60]; *survival* (version 3.2-11)[61,62]; *gridExtra* (version 2.3)[63]; *survminer* (version 0.4.9)[64]; *doSNOW* (version 1.0.19)[65]; *foreach* (version 1.5.1)[66]; *ComplexHeatmap* (version 2.8.0)[67]; and *RColorBrewer* (version 1.1-2)[68]. Boxplots are drawn with a line at the

median, two boxes extending from the median to the 25th and 75th percentiles, and two whiskers extending no further than 1.5x the inter-quartile range (i.e. 1.5x the distance between the 25th and 75th percentiles).

Survival Analyses

Months of Overall Survival (OS) or Progression Free Survival (PFS) were calculated by taking the number of days from CAN-3110 injection to death (or progression) and dividing by 365.24/12. For the purposes of our analysis, progression was defined as the earliest time of: 1) Death; 2) First new treatment; or 3) First MRI Progression. Note that only MRI's taken at least 14 days post CAN-3110 were considered. Also note that the following therapies were excluded from consideration when determining first new treatment: Dexamethasone, Wound Exploration, CSF Wound Drain, Bevacizumab, Cranial Wound Washout, and Biopsy. Kaplan-Meier survival curves were generated using the survfit() and ggsurvplot() functions in R, which produces estimates based on the Aalen-Johansen estimator. Cox proportional hazard regression modeling was performed using the coxph() function with default arguments. Hazard ratio forest plots for the Cox model were generated using the ggforest() function.

PCR Verification of CAN-3110 in patient 014

DNA was extracted from multiple areas of interest selected using H&E stained slides. For each sample, the selected areas were microdissected from three 5um sections of paraffin embedded tissue and DNA was extracted using the QIAamp® DNA FFPE Advanced Kit (Cat# 56604, Qiagen) following the manufacturers protocol. Genomic DNA from a patient diagnosed with glioblastoma and not injected with CAN-3110 (Brain gDNA) extracted as mentioned above was used as a negative control. Wild type HSV-1 and CAN-3110 viral genomic DNA was extracted following a previously published methodology[69]. A total of 10ng of DNA were added per PCR reaction and amplified using the Luna® Universal Probe One-Step RT-qPCR Kit (Cat# E3006L, New England Biolabs) following the manufacturers protocol. Primers complementary to ICP22 (Forward: ICP22.FW 5'-gagcagcgcacgatgga-5', Reverse: ICP22. RV 5'-cccgaaacagctgattgataca-3'), rat nestin promoter (Nestin; Nestin.FW sequence 5'-gccaacccatatttgagacaca-3', Nestin.RV sequence 5'-gccctgcctccgactgt-3'), and rat nestin-Hsp68 mini promoter (Nestin-Hsp68; Nestin-Hsp68.FW sequence 5'-gagaactctcggttgcagacacct-3', Nestin-Hsp68.RV sequence 5'-gctgctcagtttggatgttcctgg-3') were used for each corresponding PCR reaction. The PCR products were run in a 3% agarose gel at 90 volts for 1 hour using Quick-Loading 100 bp DNA ladder (Cat# N0551S, New England Biolabs) and visualized by SYBR safe (**Supplementary Figure 1)**.

TCR Clonotype Analyses

Genomic DNA (gDNA) from rHGGs tumors before and after injection, when available, and from PBMCs before and at a time that was as close as possible to the time of post-CAN-3110 tumor harvest or post-mortem was prepared by using a commercially available kit and instructions with the kit (QIAamp DNA FFPE Advanced UNG Kit Cat. No. / ID: 56704). TCR clonotype data was downloaded from Adaptive Biotechnologies (Seattle, WA, USA) immunoSeq Analyzer portal and analyzed using R (version 4.1.0)[54] along with packages listed in the "Data plotting and statistical analyses" methods section above. For discovery of PBMC TCR clonotypes that were associated with overall survival, productive clonotype frequencies were aggregated on the amino acid sequence level and changes in productive frequencies were calculated as the post CAN-3110 frequency minus the pre-CAN-3110 frequency. If a clonotype was only detected in the pre or the post sample for a given patient, the productive frequency was treated as 0 for the timepoint in which that clonotype was not detected. If a clonotype was not detected in either the pre or post CAN-3110 sample for a given patient, that patient was excluded from analyses aimed at associating survival with changes in productive frequency for that particular clonotype. Associations were estimated using a generalized linear model (using the glm() function with default arguments) which modeled change in productive frequency as a function of post-CAN3110 survival time in days. Note that all patients involved in this analysis are deceased, so it was not necessary to model this association using censored survival data. FDR corrected p-values[70] were calculated using p-values for clonotypes which had a median of at

least 2 reads either pre- or post-treatment across all patients and which were observed in all 21 IDH-WT paired samples with TCR clonotype data who either died of cancer or who died of non-cancer related causes at least 1 year following CAN-3110 injection (i.e. patient 021).

Definition of TCR based metrics. Several metrics were calculated from the TCR sequencing data. These are defined as follows (Immunoseq Analyzer, Adaptive Biotechnologies, Inc):

- **T Cell Fraction**: Calculated by dividing the number of productive templates (i.e. TCRβ sequences which translate to a functional TCRβ amino acid sequence) in a sample by the total number of nucleated cells in that sample (as estimated by amplification of reference gene primers).
- **Productive Frequency**: The frequency of a given TCRβ sequence in a sample as a proportion of all productive TCRβ templates measured in that sample.
- **Productive Entropy**: Calculated by summing the Productive Frequency times the log (base 2) of the same frequency over all productive rearrangements in a sample. Higher entropy indicates greater diversity of TCRβ rearrangements.

Productive Simpson Clonality: Calculated for a sample by taking the square root of Simpson's diversity index for all productive TCRβ rearrangements in a sample. Higher values indicate lower diversity of TCRβ rearrangements.

Identification of specific T cell clonotypes (related to sub-heading "Specific public T cells link to survival" in main text and Extended Data Figure 10). TCRβ DNAseq was performed (Adaptive Biotechnologies, Inc.) to identify expanding T cell clonotypes before and after CAN-3110. The panels in **Extended Data Figure 10** are the Jaccard indices and Pearson correlation coefficient maps for all public and private TCR clonotypes in tumor and PBMC T cell clones.

Generation of bulk RNA-seq data.
Fresh tumors were collected at the biopsy before CAN-3110 injection or craniotomy for resection after CAN-3110 and stored at -80 °C freezer or in liquid nitrogen. Small pieces of tissues were homogenized in TRI Reagent (Millipore Sigma) or TRIzol reagent (Thermo Fisher), and RNA-rich aqueous phase were further treated with Direct-zol RNA miniprep kit (Zymo) or Invitrogen RNeasy kit (Qiagen) to purify total RNA. Library preparation and Illumina sequencing were performed in Psomagen. TruSeq Stranded Total RNA with Ribo-Zero kit (Illumina) was used for library construction, and Illumina NovaSeq6000 with a paired-end, 150 bp read. RNA-seq reads were aligned using Kallisto v0.42.4 (https://doi.org/10.1038/nbt.3519) to GENCODE v23 transcripts. Several transcript groups were removed from the analysis: the noncoding RNA, histone- and mitochondria-related transcripts, which resulted in 20,062 protein coding genes. Gene expression was calculated as transcripts per million (TPM) and log2-transformed (https://doi.org/10.1038/s41587-020-0546-8). ssGSEA algorithm was used to calculate gene signature scores (https://doi.org/10.1016/j.ccell.2021.04.014). Raw scores were medium scaled to (-2, 2) range. VDJ transcript quantifications were performed using MiXCR v3.0.13[71,72].

Statistics and Reproducibility
The multiplex IF panel went through many rounds of optimization on 2-3 control glioblastoma samples to ensure reproducible and reliable staining conditions. Each round of optimization was carefully reviewed by a pathologist to confirm the validity of staining. To maximize use of the patient specimens, only one slide was stained with each multiplex panel. All cases were stained during the same automated staining protocol, using the same conditions for each slide. Depending on sample availability, between 3-6 regions of interest were selected for quantitative analysis to encompass the range of staining across the sample.

For immunohistochemistry staining, each immunostain was performed once per sample and the quantification was from three ROIs per slide.

TCRβ DNA sequencing and bulk RNA sequencing were performed using a single sample for each patient/timepoint being assayed.

For PCR verification of CAN-3110 in tumor samples, each reaction was run in triplicate and one of the three replicates was sent entirely for sequencing using the Nestin-Hsp68.FW primer.

Retreatment of subject 042 as patient 054. Subject 042 in cohort 9 was re-treated as subject 054 in cohort 10, 8 months later. Since the patient's radiographic response and disease progression were markedly different between the two treatments, 042 and 054 were treated as separate data points in analyses throughout this paper, with survival time calculated from the first CAN-3110 injection for 042 and from the second CAN-3110 injection for 054. In each instance in which this occurs, it is noted both how many patients and how many interventions are being used for an analysis, with each intervention being used as a separate data point in the analysis.

End Notes

Acknowledgments: The authors would like to acknowledge all the patients and families that participated in the trial, particularly subject 021 and her family. We acknowledge assistance from Steffen Ventz, Ph.D. (University of Minnesota), Grace Park, B.S., Leah M. Seften, B.S., Kin Hoe Chow, Ph.D., Aniket Shetty, M.S., William Pisano, B.A., Emily Lapinskas, B.S., and Fiona Watkinson, B.A. (Brigham and Women's Hospital). We also acknowledge assistance in TCR analyses from Drs. David Hamm and Barbara Banbury (Adaptive Biotechnologies, Inc). We acknowledge assistance from Nathan Caffo, Ph.D., Jessica Dwyer, B.S, Doug Lane, B.S., Haley White, B.S. (Candel Therapeutics, Inc.)

Funding:

National Cancer Institute P01 CA163205 (EAC/IHS/MX/ASY/ HS/ KL)
National Cancer Institute P01 CA236749 (EAC/GF/ MS/ KW/DAR)
National Institutes of Health R01NS110942 (EAC/HN)
The Sandra Jelin Plouffe Fund to Advance Glioblastoma Research (EAC)
The Oligodendroglioma Fund (EAC)
The Oppenheimer Tiger Fund (DAR)
The Daniel E. Ponton Fund (EAC)
The MIT Koch Institute Bridge Grant (EAC/ DAR)
Alliance for Cancer Gene Therapy (EAC)
KDDI Foundation Scholarship (NS)
National Institutes of Health 2T32CA079443 (ALL)
National Institutes of Health T32CA251062 (JKW)
The Leukemia and Lymphoma Society 2322-19 (LN)
Clinical Trial Regulatory Support (in-kind and financial), Candel Therapeutics, Inc.

Author contributions:

Conceptualization: EAC
Methodology: ALL, IHS, AML, HN, JKW, AS, NM, GF, XM, AYS, JG, AZ, JDB, ET, HI, JL, NS, MON, PH, RP, HS, BS, WLB, PP, EC, SWM, MCP, YZ, AC, SWR, KK, KK, ET, NM, DT, NS, AG, AS, FF, EA, LKA, JW, DK, AM, CM, PPT, FB, DK, IT, MLS, KWW, KL, DAR, EAC

Investigation: ALL, IHS, AML, HN, JKW, AS, NM, GF, XM, AYS, JG, AZ, JDB, ET, HI, JL, NS, MON, DT, PH, RP, HS, BS, WLB, PP, SWM, MCP, YZ, AC, SJR, PYW, EQL, LN, UC, LNGC, SDD, TB, KK, ET, NM, DT, NS, AG, AS, FF, EA, LKA, DK, JW, AM, CM, PPT, FB, DK, IT, MLS, KWW, KL, DAR, EAC
Visualization: ALL, IHS, AML, HN, AS, GF, XM, NS, SWM, MCP, ET, NM, DT, NS, AG, AS, FF, FB, KL, EAC
Funding acquisition: ALL, JKW, NS, EA, PPT, MLS, KWW, KL, DAR, EAC
Project administration: HN, SJR, PYW, TB, NS, EA, PPT, FB, IT, MLS, KWW, KL, DAR, EAC
Supervision: IHS, HN, GF, XM, SJR, PYW, TB, NS, PPT, FB, EA, LKA, MLS, KWW, IT, KL, DAR, EAC
Writing – original draft: ALL, EAC
Writing – review & editing: ALL, IHS, AML, HN, JKW, GF, XM, JDB, MCP, PYW, LNGC, NS, EA, LKA, PPT, FB, DK, MLS, KWW, DAR, EAC

Competing interests:

ALL, HIS, AML, HN, JKW, AS, NM, GF, XM, ASY, JG, AZ, ET, HI, JL, NS, MON, DT, PH, RP, HS, BS, EC, SWM, MCP, YZ, AC, UC, SDD, DK, KL do not have competing interests.

JDB has an equity position in Treovir Inc., an oHSV clinical stage company and is a member of the POCKiT Diagnostics Board of Scientific Advisors. JDB also has a Provisional Patent App No. 63/273,577 entitled, "METHODS AND FORMULATIONS RELATED TO THE INTRATHECAL DELIVERY OF ONCOLYTIC VIRUSES".

WLB consulted for Stryker, Inc.

PP is cofounder and member of the board of directors of Ternalys Therapeutics. He is also a named inventor on patents related to noncoding RNAs technology.

PYW received research support from Astra Zeneca/Medimmune, Beigene, Celgene, Chimerix, Eli Lily, Genentech/Roche, Kazia, MediciNova, Merck, Novartis, Nuvation Bio, Puma, Servier, Vascular Biogenics, VBI Vaccines and served on advisory boards for Astra Zeneca, Bayer, Black Diamond, Boehringer Ingelheim, Boston Pharmaceuticals, Celularity, Chimerix, Genenta, Glaxo Smith Kline, Karyopharm, Merck, Mundipharma, Novartis, Novocure, Nuvation Bio, Prelude Therapeutics, Sapience, Servier, Sagimet, Vascular Biogenics, VBI Vaccines and Data Safety Monitoring Committees for Day One Bio, and Novocure.

LN serves as a consultant for Ono and Brave Bio.

LNGC received research support from Merck (to the Dana-Farber Cancer Institute). He also has received research support from NIH, the American Society of Clinical Oncology, and the Robert Wood-Johnson Foundation.

EQL receives Royalties from Wolter Kluwer (Up to Date, Inc) and consulting fees from GCAR

TB receives clinical trial support from ONO Pharmaceuticals, publishing royalties from UpToDate, Inc and Oxford University Press

SJR receives research support from Bristol-Myers-Squibb and KITE/Gilead. SJR is on the SAB for Immunitas Therapeutics.

KK is employed by and owns equity in Clearpoint, Inc.

ET, NM, DT, NS, AG, AS, and FF are employed by BostonGene, Corp and have equity options in BostonGene, Corp.

EA is a founder, board member of, and holds equity in Candel Therapeutics

LKA is co-founder and holds equity in Candel Therapeutics

DK, JW, AM, CM, PPT, FB are employees of and hold equity in Candel Therapeutics, Inc.

IT is an advisory board member of Immunitas Therapeutics

MLS and KWW are equity holders, scientific co-founders, and advisory board members of Immunitas Therapeutics.

DAR is an advisor to Agios; AnHeart Therapeutics; Avita Biomedical, Inc., Blue Rock Therapeutics; Bristol Myers Squibb; Boston Biomedical; CureVac AG; Del Mar Pharma; DNAtrix; Hoffman-LaRoche, Ltd; Imvax;

Janssen; Kiyatec; Medicenna Therapeutics; Neuvogen; Novartis; Novocure; Pyramid; Sumitomo Dainippon Pharma; Vivacitas Oncology, Inc Y-mabs Therapeutics
EAC is an advisor to Amacathera, Bionaut Labs, Genenta, inc., Insightec, Inc., DNAtrix Inc, Seneca Therapeutics, Theravir. He has equity options in Bionaut Laboratories, DNAtrix, Immunomic Therapeutics, Seneca Therapeutics, Ternalys Therapeutics. He is co-founder and on Board of Directors of Ternalys Therapeutics. He

Patents related to oHSV and CAN-3110 are under the possession of Brigham and Women's Hospital (BWH) with EAC and HN names as co-inventors. These patents have been licensed to Candel Therapeutics, Inc. Present and future milestone license fees and future royalty fees are distributed to BWH from Candel.

Additional information:

Correspondence and reprint requests should be addressed to: E. Antonio Chiocca, MD PhD, Department of Neurosurgery, Hale Building for Transformative Medicine, 4th floor, Brigham and Women's Hospital, 60 Fenwood Street, Boston, MA 02116. Email:eachiocca@bwh.harvard.edu

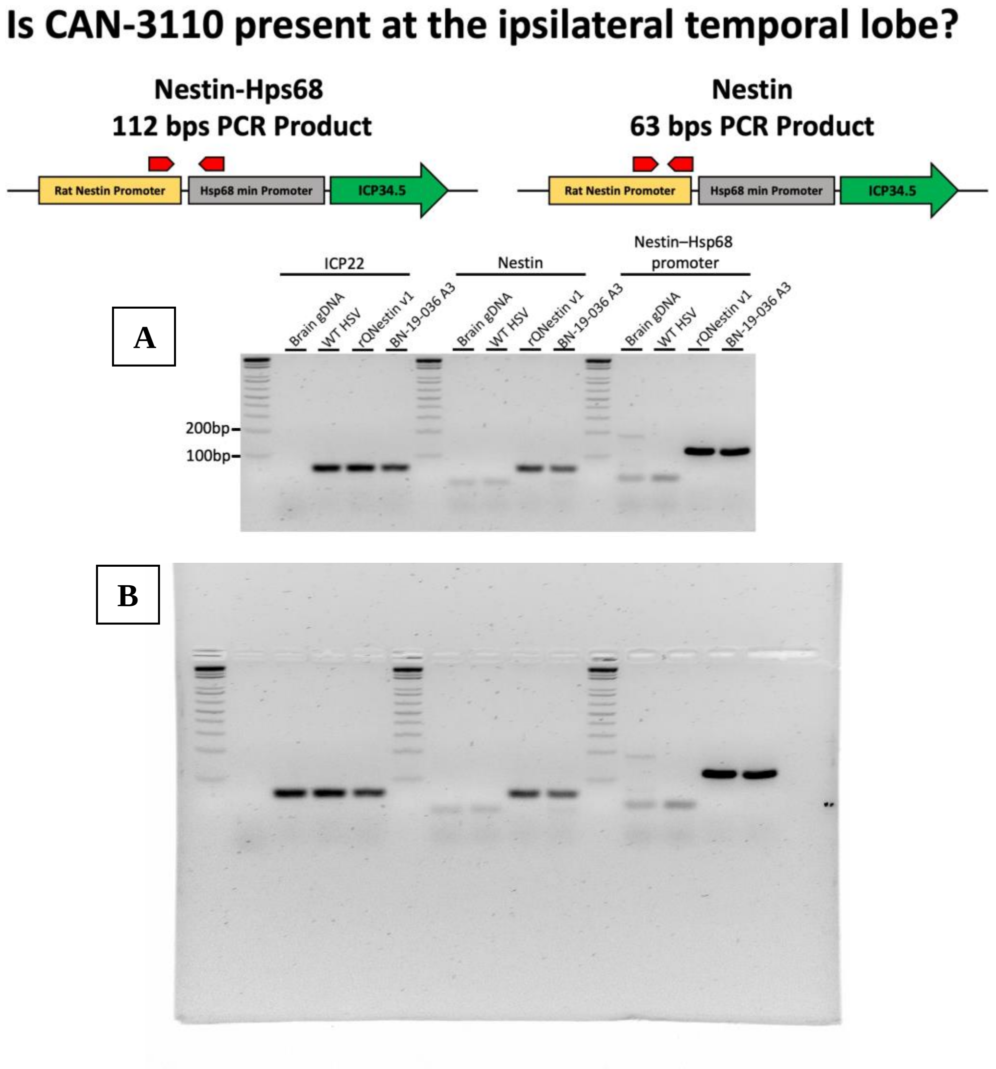

Supplementary Figure 1. Uncropped gel from Extended Data Figure 8b. (a) Image showing all samples run on the gel from **Extended Data Figure 8b**, along with sample descriptions for each lane. **(b)** Full uncropped gel from **Extended Data Figure 8b** and panel **a** of this figure.

Supplementary References

50. Delgado-López, P. D., Riñones-Mena, E. & Corrales-García, E. M. Treatment-related changes in glioblastoma: a review on the controversies in response assessment criteria and the concepts of true progression, pseudoprogression, pseudoresponse and radionecrosis. *Clin Transl Oncol* **20**, 939–953 (2018).
51. Keskin, D. B. *et al.* Neoantigen vaccine generates intratumoral T cell responses in phase Ib glioblastoma trial. *Nature* **565**, 234–239 (2019).
52. Garcia, E. P. *et al.* Validation of OncoPanel: A Targeted Next-Generation Sequencing Assay for the Detection of Somatic Variants in Cancer. *Archives of Pathology & Laboratory Medicine* **141**, 751–758 (2017).
53. Cheng, D. T. *et al.* Memorial Sloan Kettering-Integrated Mutation Profiling of Actionable Cancer Targets (MSK-IMPACT). *J Mol Diagn* **17**, 251–264 (2015).
54. R Core Team. R: A Language and Environment for Statistical Computing. Version 4.2.1 (2022) (available at https://www.R-project.org/).
55. RStudio Team. RStudio: Integrated Development for R. Version 2022.07.2 (2022) (available at http://www.rstudio.com/).
56. Schauberger, P. & Walker, A. openxlsx: Read, Write and Edit xlsx Files. Version 4.2.5 (2021) (available at https://CRAN.R-project.org/package=openxlsx).
57. Wickham, H. ggplot2: Elegant Graphics for Data Analysis. Version 2_3.3.5 (2016) (available at https://ggplot2.tidyverse.org).
58. Wickham, H. *et al.* Welcome to the Tidyverse. *Journal of Open Source Software* **4**, 1686 (2019).
59. Kassambara, A. rstatix: Pipe-Friendly Framework for Basic Statistical Tests. Version 0.7.0 (2021) (available at https://CRAN.R-project.org/package=rstatix).
60. Kassambara, A. ggpubr: ‘ggplot2’ Based Publication Ready Plots. Version 0.4.0 (2020) (available at https://CRAN.R-project.org/package=ggpubr).

61. Therneau, T. M. A Package for Survival Analysis in R. Version 3.2-11 (2021) (available at https://CRAN.R-project.org/package=survival).

62. Therneau, T. M. & Grambsch, P. M. Expected Survival. in *Modeling Survival Data: Extending the Cox Model* (eds. Therneau, T. M. & Grambsch, P. M.) 261–287 (Springer, 2000). doi:10.1007/978-1-4757-3294-8_10.

63. Auguie, B. gridExtra: Miscellaneous Functions for 'Grid' Graphics. Version 2.3 (2017) (available at https://CRAN.R-project.org/package=gridExtra).

64. Kassambara, A. survminer: Drawing Survival Curves using 'ggplot2'. Version 0.4.9 (2021) (available at https://CRAN.R-project.org/package=survminer).

65. Microsoft & Weston, S. doSNOW: Foreach Parallel Adaptor for the 'snow' Package. Version 1.0.19 (2020) (available at https://CRAN.R-project.org/package=doSNOW).

66. Microsoft & Weston, S. foreach: Provides Foreach Looping Construct. Version 1.5.1 (2020) (available at https://CRAN.R-project.org/package=foreach).

67. Gu, Z., Eils, R. & Schlesner, M. Complex heatmaps reveal patterns and correlations in multidimensional genomic data. *Bioinformatics* **32**, 2847–2849 (2016).

68. Neuwirth, E. RColorBrewer: ColorBrewer Palettes. Version 1.1.2 (2014) (available at https://CRAN.R-project.org/package=RColorBrewer).

69. Szpara, M. L., Tafuri, Y. R. & Enquist, L. W. Preparation of viral DNA from nucleocapsids. *J Vis Exp* 3151 (2011) doi:10.3791/3151.

70. Benjamini, Y. & Hochberg, Y. Controlling the False Discovery Rate: A Practical and Powerful Approach to Multiple Testing. *Journal of the Royal Statistical Society. Series B (Methodological)* **57**, 289–300 (1995).

71. Bolotin, D. A. *et al.* MiXCR: software for comprehensive adaptive immunity profiling. *Nat Methods* **12**, 380–381 (2015).

72. Bolotin, D. A. *et al.* Antigen receptor repertoire profiling from RNA-seq data. *Nat Biotechnol* **35**, 908–911 (2017).
