# Supplementary material for: Clinical trial links oncolytic immunoactivation to survival in glioblastoma
Source: Nature. 2023 Oct 18;623(7985):157–66. doi: 10.1038/s41586-023-06623-2 (PMC10620094; doi:10.1038/s41586-023-06623-2)
Supplement: Supplementary file 2 — Reporting Summary [file 41586_2023_6623_MOESM2_ESM.pdf]

nature portfolio

Corresponding author(s): E. Antonio Chiocca

Last updated by author(s): 7/31/2023

Reporting Summary

Nature Portfolio wishes to improve the reproducibility of the work that we publish. This form provides structure for consistency and transparency in reporting. For further information on Nature Portfolio policies, see our Editorial Policies and the Editorial Policy Checklist.

Statistics

For all statistical analyses, confirm that the following items are present in the figure legend, table legend, main text, or Methods section.

n/a	Confirmed	
☐	☒	The exact sample size (*n*) for each experimental group/condition, given as a discrete number and unit of measurement
☐	☒	A statement on whether measurements were taken from distinct samples or whether the same sample was measured repeatedly
☐	☒	The statistical test(s) used AND whether they are one- or two-sided<br>*Only common tests should be described solely by name; describe more complex techniques in the Methods section.*
☐	☒	A description of all covariates tested
☐	☒	A description of any assumptions or corrections, such as tests of normality and adjustment for multiple comparisons
☐	☒	A full description of the statistical parameters including central tendency (e.g. means) or other basic estimates (e.g. regression coefficient) AND variation (e.g. standard deviation) or associated estimates of uncertainty (e.g. confidence intervals)
☐	☒	For null hypothesis testing, the test statistic (e.g. *F*, *t*, *r*) with confidence intervals, effect sizes, degrees of freedom and *P* value noted<br>*Give P values as exact values whenever suitable.*
☒	☐	For Bayesian analysis, information on the choice of priors and Markov chain Monte Carlo settings
☒	☐	For hierarchical and complex designs, identification of the appropriate level for tests and full reporting of outcomes
☐	☒	Estimates of effect sizes (e.g. Cohen's *d*, Pearson's *r*), indicating how they were calculated

Our web collection on statistics for biologists contains articles on many of the points above.

Software and code

Policy information about availability of computer code

Data collection	Medical record data was collected using EPIC (v May2022) and InForm (v2.3). Immunofluorescence image acquisition was performed using the Mantra multispectral imaging platform (Vectra 3, PerkinElmer)
Data analysis	Cell identification for multiplexed immunofluorescence was performed using Akoya Inform Automated Image Analysis Software version 2.4.8. Oncoprint genomic profiling was analyzed using R 4.2.1, RStudio 2022.07.2+576, and the Oncoprint function of the ComplexHeatmap 2.12.1 package. Data plotting and statistical analyses were performed using R (version 4.1.0) and RStudio (version 2022.2.3.492) along with the following packages: openxlsx (version 4.2.5); ggplot2 (version 2_3.3.5); tidyverse (version 1.3.1); rstatix (version 0.7.0); ggpubr (version 0.4.0); survival (version 3.2-11); gridExtra (version 2.3); survminer (version 0.4.9); doSNOW (version 1.0.19); foreach (version 1.5.1); ComplexHeatmap (version 2.8.0); and RColorBrewer (version 1.1-2). RNA-seq reads were aligned using Kallisto v0.42.4. ssGSEA algorithm was used to calculate gene signature scores (https://doi.org/10.1016/j.ccell.2021.04.014). MIXCR v.3.0.13 was used to analyze T and B cell receptor repertoire from the RNA-seq samples. MRI segmentations were performed manually using 3D Slicer for cohorts 1-9 or using SmartBrush Software (version 3.0.0.92, BrainLab AG, Munich, Germany) for cohort 10.

For manuscripts utilizing custom algorithms or software that are central to the research but not yet described in published literature, software must be made available to editors and reviewers. We strongly encourage code deposition in a community repository (e.g. GitHub). See the Nature Portfolio guidelines for submitting code & software for further information.

Data

Policy information about availability of data

All manuscripts must include a data availability statement. This statement should provide the following information, where applicable:

- Accession codes, unique identifiers, or web links for publicly available datasets
- A description of any restrictions on data availability
- For clinical datasets or third party data, please ensure that the statement adheres to our policy

Patient responses, demographic information, and safety outcomes, as well IHC quantifications and RNAseq gene signature scores are available within the paper and its Supplementary Information. Raw RNA sequencing and TCRβ DNA sequencing files have been deposited in a controlled access repository at the database of Genotypes and Phenotypes (dbGaP): http://www.ncbi.nlm.nih.gov/projects/gap/cgi-bin/study.cgi?study_id=phs003378.v1.p1

Human research participants

Policy information about studies involving human research participants and Sex and Gender in Research.

Reporting on sex and gender	Biological gender was included in a CoxPH multivariate analysis of post-treatment survival alongside other potential covariates of survival. Gender was not determined to be a significant factor in patient survival following therapy in this trial, and, given the small sample size available in a phase I trial, no further analyses of gender were performed.
Population characteristics	Population characteristics are fully described in Extended Data Supplementary Tables 1A-1C of the manuscript.
Recruitment	Potentially eligible subjects were recruited from: 1- subjects seen or referred to our brain tumor clinics at Dana-Farber Cancer Institute and Brigham and Women's Hospital, 2- subjects made aware of the study via the clinicaltrials.gov website, 3- subjects referred from national patient referral organizations such as the National Brain Tumor Consortium, 4- subjects referred by direct physician or other healthcare professional, 5- subjects made aware via word of mouth, or via personal searches. Bias in patient selection is always possible. Potentially eligible patients were accrued by internal referral, by external referrals, by patients seeking care after finding out about the trial or by patient care clinical trial networks referring patients for consideration into the trial. To minimize bias, an independent neurosurgeon, external to our institution (Dr. Ekkehard Kasper, St. Elizabeth's Medical Center, Boston MA) reviewed eligibility for each patient's MRIs, history, medical exams before proceeding with the trial.
Ethics oversight	This phase 1 clinical trial was reviewed and approved by NIH RAC Office of Biotechnology Affairs (NIH no 1104-1100) and the IRB from the DFCI (no 16-557). The IND Sponsor was Dr. Chiocca (IND 16380).

Note that full information on the approval of the study protocol must also be provided in the manuscript.

Field-specific reporting

Please select the one below that is the best fit for your research. If you are not sure, read the appropriate sections before making your selection.

☒ Life sciences ☐ Behavioural & social sciences ☐ Ecological, evolutionary & environmental sciences

For a reference copy of the document with all sections, see nature.com/documents/nr-reporting-summary-flat.pdf

Life sciences study design

All studies must disclose on these points even when the disclosure is negative.

Sample size	Since this was a 3+3 dose escalation phase I trial, sample sizes for cohorts 1-9 were defined by the dose-escalation schema and were not sized to obtain statistical power for correlative analyses. As such, no statistical methods were used to pre-determine sample sizes. Sample size for cohort 10 was based on feasibility as a small exploratory expansion cohort, again, with no statistical power analyses being performed when selecting sample size in this phase I trial. In this exploratory analysis, samples sizes for immunohistochemistry, TCRbeta sequencing, and RNAsequencing were dictated by the availability of high-quality tissues for staining/DNA or RNA extraction.
Data exclusions	As clearly stated in all relevant analyses, patient 045 was excluded from analyses due to having experienced a non-GBM mortality shortly after trial enrollment. This exclusion criteria was not established prior to trial enrollment; however, most analyses were never performed in a way that included patient 045, and we are unaware of any analysis which would have resulted in a different outcome had patient 045 been included.
Replication	Due to the expense and time involved with conducting clinical trial research, replication was not feasible for any of the experiments presented in this manuscript. It is our intent to replicate findings from this manuscript in later clinical trials and separate manuscripts as the data becomes available over the coming years.
Randomization	As routinely done for Phase 1 studies, randomization was not possible in this study, because only one cohort was open for recruitment at any given time.

Blinding	Blinding was not possible for patients caregivers in this study, because, given that this was a dose-escalation trial phase 1 study , only one cohort was open for recruitment at any given time. This fact also made it impractical to blind researchers to patient group when determining tumor volumes or immune infiltration. However, rigorous and consistent criteria were applied when grading patients (as described in supplemental methods), and we do not believe significant bias occurred due to this lack of blinding. This is especially true since we see concordance in the data between potentially subjective metrics of immune infiltration (i.e. pathological quantifications) and strictly quantitative metrics (i.e. ImmunoSeq quantifications).

Reporting for specific materials, systems and methods

We require information from authors about some types of materials, experimental systems and methods used in many studies. Here, indicate whether each material, system or method listed is relevant to your study. If you are not sure if a list item applies to your research, read the appropriate section before selecting a response.

Materials & experimental systems

n/a	Involved in the study
☐	☒ Antibodies
☒	☐ Eukaryotic cell lines
☒	☐ Palaeontology and archaeology
☒	☐ Animals and other organisms
☐	☒ Clinical data
☒	☐ Dual use research of concern

Methods

n/a	Involved in the study
☒	☐ ChIP-seq
☒	☐ Flow cytometry
☐	☒ MRI-based neuroimaging

Antibodies

Antibodies used	HSV-1 polyclonal antibody (Dako Polyclonal); CD4 (Dako 4B12), CD8 (Dako 144D), CD20 (Dako L26), Nestin (Cell Signaling Technologies 10C2), Nectin-1/CD111 (Santa Cruz Biotech CK6), Sox2 (Cell Signaling Technologies D6D9), CD68 (Dako PG-M1), CD163 (Novocastra 10D6), PD-L1 (Cell Signaling Technologies E1L3N). Antibody dilutions can be found it the supplemental methods.
Validation	For multiplexed immunofluorecence, all of the antibodies are commonly used. Each antibody was first optimized by standard IHC to confirm fidelity of the staining, then adapted to single-immunofluorescence staining before combining antibodies together into a multiplex immunofluorescence panel. In single-immunofluorescence, repeated rounds of optimization include testing different antigen retrieval conditions, diluents and a wide range of antibody concentrations. In multiplex, different panel conditions are tested to ensure high signal to noise for each individual marker, while eliminating bleedthrough, crosstalk between channels, and nonspecific staining.<br>For chromogenic immunohistochemistry, all staining was perfomed utilizing commercially available antibodies optimized for staining formalin-fixed paraffin-embedded tissue sections, with staining performed in a CAP certified laboratory.

Clinical data

Policy information about clinical studies

All manuscripts should comply with the ICMJE guidelines for publication of clinical research and a completed CONSORT checklist must be included with all submissions.

Clinical trial registration	NCT03152318
Study protocol	Study protocol can be accessed at: https://www.dropbox.com/s/rj035h42svm7i71/16-557%20Protocol%20Cohort%2010%20Arm%20A%2003FEB2020%20-%20clean.pdf?dl=0
Data collection	All data were collected at the Brigham and Women's Hospital and/or Dana Farber Cancer Institute. In some cases for patients who were not local, some data was collected at their outside hospital and physician place of care. Outside collections occurred for some patients who lived in Florida, New York, New Hampshire, Maine, Vermont, Connecticut, Rhode Island. Period times for recruitment were from September 2017 until December 2020. Data collection occurred between September 2017 until March 2023 when a subject of arm A underwent resection of recurrence of their high grade glioma after treatment with CAN-3110.
Outcomes	Outcome descriptions are too lengthy to include here, but are described in detail in the supplemental methods sections:<br>1. Clinical protocol: definition of adverse event (AE), serious adverse event (SAE), dose limiting toxicity (DLT), and maximum tolerated dose (MTD).<br>2. Clinical Protocol: response assessment

Magnetic resonance imaging

Experimental design

Design type	Standard MRI sequences for brain tumors (with and without gadolinium) obtained preoperatively, withing 72 hours post-operatively and then every 8 weeks. In most cases additional MRIs were available before and after intrevention

Design specifications: Standard specifications for routine clinical MRIs.

Behavioral performance measures: Behavioral performance measures were not a part of this study.

Acquisition

Imaging type(s): Regular MRI sequences for all, including perfusion imaging. If clinically indicated (close to eloquent brain), funtional imaging with tractography performed too

Field strength: 3 Tesla

Sequence & imaging parameters: T1, T2, FLAIR, T1 with gadolinium, DWI, ADC, DTI

Area of acquisition: Whole brain

Diffusion MRI: ☐ Used ☒ Not used

Preprocessing

Preprocessing software: Segmentations were performed manually by a trained neurosurgeon using 3D Slicer (v5.1.0-2022-10-31 or previous) for cohorts 1-9. or using SmartBrush Software (version 3.0.0.92, BrainLab AG, Munich, Germany) for cohort 10.

Normalization: No pre-processing was performed prior to segmentation.

Normalization template: No pre-processing was performed prior to segmentation.

Noise and artifact removal: No pre-processing was performed prior to segmentation.

Volume censoring: No pre-processing was performed prior to segmentation.

Statistical modeling & inference

Model type and settings: No statistical modeling/inference was performed using the MRI data.

Effect(s) tested: No statistical modeling/inference was performed using the MRI data.

Specify type of analysis: ☐ Whole brain ☒ ROI-based ☐ Both

Anatomical location(s): Volumes were obtained specifically for tumor regions of the brain.

Statistic type for inference (See Eklund et al. 2016): No statistical modeling/inference was performed using the MRI data.

Correction: No statistical modeling/inference was performed using the MRI data.

Models & analysis

n/a	Involved in the study
☒	☐ Functional and/or effective connectivity
☒	☐ Graph analysis
☒	☐ Multivariate modeling or predictive analysis
